# The Roll of Honour of the Royal College of Surgeons in Ireland

**Published:** 1915-01-23

**Authors:** Alfred Miller

**Affiliations:** Registrar. Dublin


### THE EDITOR'S LETTER-BOX.

### The Roll of Honour of the Royal College of Surgeons in Ireland.

*To the Editor of* The Hospital.

Sir,—May I through the medium of your paper mention that the President, Vice-President, and Council of the Royal College of Surgeons in Ireland have decided to place in the College a permanent record of the names of all the students, licentiates, and Fellows of the College who are at present serving with His Majesty's Navy and Expeditionary Forces, and further to erect a suitable memorial to all such as fall in the war?

The President, Vice-President, and Council will be glad if the relatives and friends would communicate the names of such students, licentiates, and Fellows to the Registrar of the College.—I am, yours faithfully,

Alfred Miller, Registrar.

Dublin, January 16, 1915.

